# Contrôle, élimination, éradication : où en est-on dans les MTN ? Journée scientifique de la SFMTSI du 25 novembre 2021.

**DOI:** 10.48327/mtsi.2021.189

**Published:** 2021-12-10

**Authors:** Jacques Chandenier, Jean-Philippe Chippaux, Pierre Gazin, Jean Jannin, Philippe Solano

**Affiliations:** SFMTSI. Hôpital Pitié-Salpêtrière - Pavillon Laveran, 47-83 Boulevard de l'Hôpital, 75651 Paris cedex 13

**Keywords:** Maladies tropicales négligées, Médecine tropicale, Ulcère de Buruli, Echinococcose, Onchocercose, Maladie de Chagas, Schistosomoses, Trypanosomiase humaine africaine, Géohelminthiases, Dracunculose, Pays en développement, Neglected tropical diseases, Tropical medicine, Buruli ulcer, Echinococcosis Onchocerciasis, Chagas disease, Schistosomiasis, Human African trypanosomiasis, Geohelminthiasis, Dracunculiasis, Developing countries

Les maladies tropicales négligées (MTN) constituent un groupe de vingt maladies infectieuses hétérogènes par leur agent causal, leur technique de prévention et de traitement. Elles ont en commun d'atteindre les populations les plus pauvres, souvent rurales et isolées, des régions tropicales où le développement, quels que soient ses formes et ses choix, a le plus de difficultés à se réaliser. Facteurs de pauvreté, ces pathologies en sont également la conséquence, maintenant environ un milliard d'humains dans la souffrance. Les combattre est une nécessité pour favoriser un développement plus harmonieux, moins inégalitaire. Pour y parvenir, il faut bien connaître leur épidémiologie, les moyens de lutte pertinents, l'importance des facteurs humains et environnementaux. Différentes par leurs agents étiologiques, leurs modes de transmission, leurs moyens de lutte préventive et curative, ces pathologies ont en commun des caractéristiques favorisant leur réduction avec, pour certaines, la perspective réelle d’être durablement contrôlées voire éliminées. Des traitements efficaces, bien tolérés et d'un faible coût permettent des stratégies de lutte communautaire ou à large échelle. Ils utilisent généralement des molécules anciennes, la recherche pharmaceutique pour la mise au point de nouvelles molécules étant une activité peu rentable. La recherche publique et le financement par les pays les plus avancés ainsi que par les institutions interétatiques des Nations unies ou humanitaires doivent jouer un rôle de première importance, sous réserve d’être coordonnés. Leur contrôle passe également par l’éducation, leur compréhension par les populations concernées, avec la perspective d'améliorer leurs conditions de vie.

La Journée scientifique d'automne de la SFMTSI ce 25 novembre 2021 a été l'opportunité d'une présentation par des acteurs de terrain, de la situation actuelle d'une partie de ces maladies, les avancées et les limites actuelles d'une lutte intégrée ou non. Le choix a été fait de ne pas être exhaustif, mais de privilégier les pathologies pour lesquelles les résultats, en termes de santé publique, ont été les plus marquants au cours des dernières années.

