# Supplementary material for: Control, elimination, eradication: Where do we stand with NTDs? Scientific day of the SFMTSI, 25 November 2021
Source: Med Trop Sante Int. 2021 Dec 10;1(4):mtsi.2021.189. [Article in French] doi: 10.48327/mtsi.2021.189 (PMC9128498; doi:10.48327/mtsi.2021.189)
Supplement: Related texts

# Research article: Contrôle, élimination, eradication

**Published:** 2021-12-10

**Authors:** Jean JANNIN

**Affiliations:** SFMTSI. Hôpital Pitié-Salpêtrière - Pavillon Laveran, 47-83 Boulevard de l'Hôpital, 75651 Paris cedex 13

Les MTN sont des maladies éliminables. Le chemin de l’élimination est long et passe par différentes étapes avant d'atteindre le moment ou l’élimination peut être annoncée. Ce moment de l’élimination est éphémère et ne peut être conçu sans la notion de durabilité. Les définitions des différents stades de contrôle, élimination ou éradication sont strictement définies par l'OMS mais ne décrivent pas correctement la phase « péri » élimination dont la complexité est grande et déborde largement le cadre strictement scientifique.

La feuille de route de l'OMS décrit les objectifs de l’élimination pour les MTN dans leur diversité et si chaque étape de leur mise en œuvre est spécifique, un certain nombre de principes restent communs et doivent être respectés sous peine d’échec.

# Research article: Onchocercose

**Published:** 2021-12-10

**Authors:** Michel BOUSSINESQ

**Affiliations:** Institut de Recherche pour le Développement, UMI-233 TransVIHMI (IRD-Inserm-Université de Montpellier), Montpellier

Les traitements de masse par ivermectine (150 μg/kg en dose unique) organisés depuis 1990 dans les zones où l'onchocercose est méso- ou hyperendémique ont permis d’éliminer la maladie en tant que problème de santé publique. En 2012, l'OMS a élargi son objectif à l’élimination de l'infection et sa feuille de route pour 2021-2030 prévoit que 12 pays auront vérifié l'interruption de la transmission en 2030. D'ores et déjà, cet objectif a été atteint dans 4 pays latino-américains et quelques foyers africains (Ouganda, Soudan…). Ailleurs, les activités de lutte doivent être étendues aux zones hypoendémiques, dont les limites restent à préciser. Les outils utilisés pour cette cartographie (sérologie, recherche d'ADN parasitaire chez les vecteurs) posent quelques problèmes. Par ailleurs, en Afrique centrale, la co-endémie avec la loase interdit la distribution de masse d'ivermectine dans les zones où l'onchocercose est hypoendémique car le risque d'effet secondaire grave (ESG: encéphalopathies à Loa) y excèderait le bénéfice des traitements. Des stratégies alternatives, plus coûteuses que le traitement de masse par ivermectine, doivent donc être employées dans ces régions de co-endémie. Ainsi, des stratégies Test-and-treat visant à identifier soit les rares sujets à risque d'ESG devant être exclus du traitement par ivermectine, soit les personnes effectivement infectées par *Onchocerca volvulus* et qui doivent être traitées, ont été proposées. Une autre approche consisterait à réaliser un pré-traitement de masse des populations avec un médicament abaissant progressivement les densités microfilarienne à Loa au-dessous du seuil de risque d'apparition des ESG, puis à distribuer, sans risque, l'ivermectine. Dans les régions sans loase, des traitements de masse par ivermectine plus rapprochés (semestriels, voire trimestriels) ou par moxidectine, permettraient d'accélérer l’élimination de l'onchocercose. La moxidectine, qui abaisse les densités microfilariennes beaucoup plus longtemps que l'ivermectine, a été approuvée par la FDA en 2018 pour le traitement de l'onchocercose (8 mg en dose unique). Elle fait l'objet d'essais supplémentaires pour que l'OMS recommande son utilisation contre l'onchocercose. Par ailleurs, 3 médicaments potentiellement macrofilaricides (tuant les vers adultes) ont fait l'objet d'essais de phase 1: l’émodepside, l'oxfendazole et le TylAMac (ABBV-4083). Les essais précliniques ont montré que les 2 premiers ont un effet macrofilaricide direct sur les filaires tandis que le TylAMac élimine les bactéries symbiotiques du genre *Wolbachia* nécessaires à la survie et à la reproduction des parasites. Des essais en phase 2 sont programmés, avec des traitements répétés plusieurs jours de suite.

# Research article: Les défis de l’éradication du ver de Guinée

**Published:** 2021-12-10

**Authors:** Jean-Philippe CHIPPAUX

**Affiliations:** MERIT, Institut de Recherche pour le développement et Université de Paris, 4 Avenue de l'Observatoire, 75006 Paris, France

La maladie du ver de Guinée, ou dracunculose, est causée par l'ingestion de copépodes dulçaquicoles infectés par des larves de *Dracunculus medinensis.* Alors que la variole est la seule infection humaine à avoir été éradiquée, le Programme d’éradication de la dracunculose (PEVG) est, avec la polio, l'un des deux programmes d’éradication actifs de l'OMS.

Le ver de Guinée, particulièrement fréquent dans de nombreux pays d'Afrique subsaharienne et d'Asie jusque dans les années 80, a vu son incidence décroître considérablement grâce à l'amélioration de l'approvisionnement en eau de boisson dans les zones rurales reculées et aux interventions des programmes nationaux de lutte contre le ver de Guinée. Son incidence annuelle est passée d'environ 3,6 millions de cas humains à la fin des années 1980 à 27 en 2020.

En l'absence d'un traitement efficace, la stratégie de lutte contre la dracunculose est basée sur des mesures de santé publique telles que l'accès à l'eau potable, l’éducation, une surveillance renforcée, le confinement des cas, le contrôle des copépodes et des incitations à la notification des cas. Il n'existe aucun médicament ni vaccin utilisable en prophylaxie ou traitement de la maladie. Seize pays ont déjà été certifiés exempts de transmission de *D. medinensis* et 2 sont en attente de l’être. Cinq pays restent endémiques: l'Angola, l’Éthiopie, le Mali, le Soudan du Sud et le Tchad.

Cependant, le programme est confronté à des défis majeurs à mesure qu'il progresse vers l’éradication, car la certification de l'absence mondiale de transmission de l'agent pathogène nécessite le respect de critères stricts. Les problèmes actuels concernent: a) des ressources insuffisantes ou mal utilisées; b) une insécurité récurrente empêchant la poursuite des interventions de contrôle de la maladie et le travail des équipes internationales de certification; c) des migrations de populations d'un pays endémique vers d'autres certifiés exempts de transmission, notamment les pays limitrophes; d) l'appauvrissement des populations qui réduit l'entretien et l'usage des ouvrages de distribution d'eau; e) l'existence d'un cycle domestique animal (notamment canidés) ou d'hôtes paraténiques (poissons, batraciens) susceptibles de maintenir le parasite; f) l'infection humaine par une espèce distincte mais voisine de *D. medinensis.*

Ces contraintes expliquent, à des degrés divers, le retard important que connaît l’éradication du ver de Guinée.

# Research article: Hommage à Philippe RANQUE

**Published:** 2021-12-10

**Authors:** Hubert BALIQUE

**Affiliations:** Retraité d'Aix Marseille Université

Résumé non communiqué.

# Research article: Lutte contre l'ulcère de Buruli en Afrique: État des lieux et perspectives

**Published:** 2021-12-10

**Authors:** Ghislain SOPOH

**Affiliations:** IRSP, Bénin

L'ulcère de Buruli (UB), causé par *Mycobacterium ulcerans,* est une maladie tropicale négligée avec des manifestations cutanées polymorphes. La grande variabilité de la présentation clinique et le large éventail de conditions similaires rendent la gestion difficile, en particulier dans les pays en développement, où les cliniciens doivent souvent travailler avec un soutien de laboratoire limité. Par ailleurs, le mode de transmission n'est pas totalement établi, mais des études ont démontré que le contact prolongé et répété des populations avec les sources naturelles d'eau, à la recherche de l'eau pour le ménage, augmente la probabilité de contracter la maladie.

Bien qu'il existe désormais des tests de diagnostic performants, les cibles de l'Organisation mondiale de la santé (OMS) peinent à être atteintes par les pays, du fait de la grande variété du diagnostic différentiel de la maladie, mais aussi, de la faible maîtrise des critères du diagnostic clinique. Par ailleurs, l’épidémiologie de la maladie semble ne pas avoir encore été bien maîtrisée, avec un descriptif très variable, probablement dépendant des activités de lutte. Récemment, l'OMS a développé un algorithme de diagnostic clinique, sous la forme de scores basés sur les connaissances épidémiologiques et cliniques existantes. De même, dans les pays où l'ampleur de la maladie évolue de manière décroissante, la mise en œuvre d'une stratégie intégrée de lutte, à divers niveaux donne des résultats intéressants, en termes d'efficience et d'impact sur plusieurs maladies négligées et sur le système de santé. Les expériences rapportées au Bénin, et ailleurs, montrent en effet, qu’à travers la mise en œuvre d'une activité de lutte intégrée, plusieurs maladies tropicales négligées à manifestation cutanée, telles que la scabiose, le mycétome et autres mycoses profondes, mais aussi la bilharziose, sont dépistées et prises en charge. Les perspectives de la lutte contre l'ulcère de Buruli passent par une meilleure connaissance de ses manifestations cliniques par les agents de santé de première ligne, la disponibilité de tests de dépistage rapides fiables ainsi que la promotion de l'accès à l'eau potable, l'hygiène et l'assainissement du milieu, la prise en charge communautaire des plaies et la mise en œuvre d'interventions de dépistage précoce intégrées.

# Research article: Trypanosomiase humaine africaine: Une bataille de gagnée mais la lutte continue!

**Published:** 2021-12-10

**Authors:** Dramane KABA, Vincent JAMONNEAU

**Affiliations:** 1 Institut Pierre Richet(IPR) de Bouaké, Centre de recherche de l'Institut national de santé publique de Côte d'Ivoire (INSP); 2 IRD Bobo Dioulasso, 01 BP 171, Bobo Dioulasso. Burkina Faso

La trypanosomiase humaine africaine (THA) est une affection parasitaire causée par un trypanosome transmis à l'Homme par la glossine ou mouche tsé-tsé. Si les efforts considérables de lutte menés dans la première moitié du 20^e^ siècle ont permis d'endiguer une épidémie qui a fait plusieurs millions de morts dans les années 1920 et 1930, et de déclarer la maladie « résiduelle » dans les années 1960, le relâchement qui a suivi, dans un contexte de nouvelles interactions entre l'homme et son environnement, a été responsable d'une réémergence, puis d'une situation épidémique de nouveau très meurtrière dans les années 1990. Grâce à une riposte efficace qui s'est organisée autour des structures nationales dédiées à la lutte contre la maladie, avec le soutien de l'OMS et l'appui de la recherche, le nombre de cas rapportés annuellement est passé en 2018 sous la barre des 1 000 pour la première fois de l'histoire.

Les stratégies de lutte contre la THA visent à dépister, traiter les cas et à lutter contre la glossine vectrice pour limiter le risque de transmission. En passant en 20 ans d'un contexte épidémique à un contexte d’élimination, la maladie du sommeil a subi une transition épidémiologique sans précédent à laquelle les stratégies et les outils de lutte ont dû s'adapter.

Ceci a été le cas en Côte d'Ivoire où un appui important de la recherche multidisciplinaire au service du Programme national d’élimination a permis de combattre la maladie sur tous les fronts. Les résultats spectaculaires obtenus avec seulement 9 cas rapportés entre 2015 et 2019 ont permis de valider l’élimination de la THA comme problème de santé publique en décembre 2020.

Il est aujourd'hui essentiel de poursuivre les actions de prévention et de prise en charge dans un objectif de durabilité. Pour cela, il est crucial de prendre en compte à la fois (i) les facteurs épidémiologiques comme le réservoir résiduel humain et le réservoir animal du trypanosome pathogène pour l'homme; (ii) les facteurs sociologiques dans un contexte où les populations et les personnels de santé vont de moins en moins considérer la maladie comme une menace et (iii) les facteurs environnementaux pour la prise en compte de l'effet des changements globaux sur les conditions de transmission locale.

Les auteurs mettront l'accent sur ces nouveaux défis à relever pour atteindre l'interruption de la transmission fixée par l'OMS en 2030.

# Research article: Echinococcose

**Published:** 2021-12-10

**Authors:** Bernadette ABELA-RIDDER

**Affiliations:** Compte-rendu par Jean-Paul BOUTIN

Alors que certaines maladies tropicales négligées (MTN) en sont aujourd'hui aux étapes d’élimination voire d’éradication, le problème est tout autre pour la lutte contre l’échinococcose pour laquelle l'OMS espère qu'au rendez-vous de sa Feuille de route 2021-2030, au moins 17 pays hyperendémiques (prévalence supérieure à 5 cas pour 100 000 habitants) auront un programme de contrôle efficient. En effet, comparée à d'autres MTN, la lutte contre l’échinococcose, véritable problème de santé humaine, animale et économique, souffre de plusieurs déficits qui entravent la mise en place de programmes nationaux ou régionaux de lutte.

C'est d'abord l'absence de systèmes de surveillance épidémiologique, humaine et animale, de qualité, et aussi l'absence de moyens diagnostics de terrain rapides, accessibles et validés, le défaut d'accès aux services par défaut de planification, de gouvernance, d’évaluation, de moyens humains et enfin financiers. Finalement, le seul axe positif à ce jour est la disponibilité de traitements humains et animaux à l'efficacité prouvée, ainsi que d'un vaccin de qualité pour le cheptel ovin. Par ailleurs, la lutte contre l’échinococcose pâtie du très long délai entre infection et manifestation clinique et du caractère très focal de son épidémiologie, de la variabilité des stratégies mises en œuvre sans coordination et de l'absence d'un véritable continuum d'intérêts entre éleveurs et consommateurs. Pour lutter contre ce fléau mondial véritable défi à la stratégie One Health, l'OMS, la FAO et l'OIE déjà engagés dans une collaboration tripartite seront prochainement rejoints par le PNUE.

Pourtant il existe des stratégies prometteuses comme la chimioprophylaxie itérative des chiens par le praziquantel, la vaccination du cheptel ovin à condition que l’éleveur en tire profit, ou l’éducation à l'hygiène alimentaire. Les programmes de contrôle pourraient s'intégrer avantageusement à ceux déjà existants de lutte contre la peste porcine, la brucellose, la rage canine, ou d’éradication de la peste des petits ruminants; et encore être intégrés au dépistage du cancer du foie chez l'Homme ou à ceux de contrôle des viandes.

Dans ce contexte, l'OMS axe son action dans l'appui aux programmes nationaux comme en Mongolie, aux coordinations régionales comme en Amérique du Sud ou en Asie centrale et au Caucase, à l’élaboration et à la diffusion de documentations pédagogiques en de nombreuses langues. Par ailleurs l'OMS convoque et anime un groupe de travail d'experts en particulier pour le prochain round sur la thématique du diagnostic de terrain (normalisation d'un sérodiagnostic des coproantigènes canins, d'un sérodiagnostic humain) et sur celui de la disponibilité de l'albendazole générique en médecine humaine. L'OMS cherche aussi à faire évoluer la CIM-11 pour qu'y soit dorénavant différenciée l'hydatidose de l’échinococcose alvéolaire, une étape préalable à l'amélioration des systèmes de surveillance. Enfin l'OMS voudrait faire naître une donation à l'exemple de ce qui est connu pour d'autres MTN.

# Research article: Le progrès de la lutte contre les géohelminthiases jusqu'en 2020, et les objectifs pour 2030

**Published:** 2021-12-10

**Authors:** Antonio MONTRESOR, Albis GABRIELLI

**Affiliations:** OMS

La présentation a abordé les sujets suivants: introduction aux géo-helminthiases; leur distribution géographique; groupes de population à risque; stratégies de lutte; élimination de la morbidité par la chimiothérapie préventive; assainissement; progrès dans la couverture des interventions de chimiothérapie préventive entre 2008 et 2020; le défi représenté par les femmes en âge de procréer; les années de vie ajustées sur l'incapacité (DALYs); le risque du développement de la résistance aux médicaments; la strongyloïdose et son inclusion parmi les géo-helminthiases; les objectifs OMS pour 2030.

# Research article: Maladie de Chagas. Vérifier les avances, briser le silence épidémiologique et mettre en œuvre un nouvel agenda de travail ambitieux

**Published:** 2021-12-10

**Authors:** Pedro ALBAJAR-VINAS

**Affiliations:** OMS

L'Organisation mondiale de la santé estime qu'environ 6 à 7 millions de personnes dans le monde sont infectées par *Trypanosoma cruzi,* le parasite qui cause l'une des maladies tropicales les plus négligées. Des cas humains de cette infection ont été diagnostiqués dans 42 pays sur 6 continents. La France est un pays endémique pour la maladie de Chagas. En effet, bien que le parasite *T. cruzi* ne soit pas présent en France métropolitaine, il l'est en Guyane française. La punaise pouvant être porteuse du parasite *T. cruzi* est également présente sur l’Île de la Réunion. Une des dernières études a estimé qu'en France métropolitaine il y aurait 156 895 personnes à risque et 1 464 personnes infectées.

De nombreuses avancées dans la lutte contre les insectes vecteurs en Amérique latine et la transfusion de sang dans 5 continents ont déjà été possibles. La disponibilité des nouveaux kits de diagnostic et les médicaments avec nouvelles présentations pédiatriques sont une réalité. Aussi, certains objectifs ont été déjà atteints en permettant un impact plus important sur la santé publique: intégration systématique de la dimension psychosociale (approche multidimensionnel); Journée mondiale de la Maladie de Chagas; feuille de route pour les maladies tropicales négligées 2021-2030; réseau de centres sentinelles de pharmaco-épidémiologie; approbation de grands projets (IntegraChagas, projet d'UNITAID sur la maladie de Chagas congénitale, Programme ibéro-américain d’élimination de la maladie de Chagas congénitale); croissance du nombre d'associations de personnes affectées par la maladie de Chagas (plus de 30 sur 5 continents), fédération internationale.

De plus, la maladie de Chagas est très liée à des déterminants sociaux (urbanisation, migration et santé, modification des pyramides populationnelles) et environnementaux (réchauffement climatique, destruction du milieu naturel). Le contexte actuel, avec la syndémie causée par la Covid-19, a déjà modifié la population à risque et le profil épidémiologique de la maladie.

La détection précoce des cas aigus et chroniques, la prise en charge et le suivi des personnes affectées, la notification des cas chroniques et le suivi épidémiologique restent les grands défis. La nouvelle ère numérique et les nombreuses avancées technologiques sont en train d'ouvrir de nouvelles opportunités de détection et de contrôle de la transmission. Le système d'information open-source et big data de l'OMS a initié la collecte de données épidémiologiques (généralement dispersées en dehors des ministères de la Santé), mais l'objectif de briser définitivement le silence épidémiologique sur la maladie de Chagas est une tâche pour absolument tous les acteurs sociaux (facteur humain).

# Research article: Nouvelle feuille de route pour la lutte contre les maladies tropicales négligées: Contrôle et élimination des schistosomoses

**Published:** 2021-12-10

**Authors:** Amadou GARBA DJIRMAY

**Affiliations:** OMS

La schistosomose humaine est causée essentiellement par 6 espèces de trématodes: *S. haematobium, S. mansoni,* S. *intercalatum, S. guineensis, S. japonicum,* et *S. mekongi.* Les espèces prédominantes sont *S. haematobium* et *S. mansoni.* Les groupes plus particulièrement exposés au risque de schistosomose sont les enfants d’âge préscolaire, les enfants d’âge scolaire, les adultes appartenant à certaines catégories professionnelles, les femmes qui sont au contact d'eau infestée dans le cadre de leurs tâches ménagères et des communautés entières dans les zones à haut risque.

La maladie est endémique dans 78 pays parmi lesquels 51 nécessitent la mise en œuvre de chimioprévention, qui est la stratégie de l'OMS consistant en l'administration périodique de praziquantel aux groupes à risque, pour juguler la morbidité liée à la schistosomose.

À l’échelle mondiale, plus de 240 millions de personnes ont besoin d'une chimioprevention contre la schistosomose parmi lesquels 90% vivent en Afrique.

En janvier 2021, l'OMS a lancé une nouvelle feuille de route 2021-2030 pour les maladies tropicales négligées. Ce document définit les cibles et les jalons au niveau mondial pour prévenir, contrôler, éliminer ou éradiquer 20 maladies et groupes de maladies tropicales négligées afin de contribuer à l'atteinte des objectifs de développement durable.

L'exposé a présenté la situation mondiale de la schistosomose, les objectifs fixés par la nouvelle feuille de route ainsi que les défis qu'ils faudra relever pour l'atteinte des objectifs et leur pérennisation.
